# Bioefficacy of Composite Medicinal Plant Extracts and Gum Arabic on Improving Postharvest Quality in Dragon Fruit

**DOI:** 10.1155/2022/3889563

**Published:** 2022-10-25

**Authors:** Paa Kwesi Bordoh, Asgar Ali, Matthew Dickinson, Yasmeen Siddiqui, Francisca Aba Ansah

**Affiliations:** ^1^Centre of Excellence for Postharvest Biotechnology (CEPB), School of Biosciences, The University of Nottingham Malaysia, Jalan Broga, 43500 Semenyih, Selangor D.E., Malaysia; ^2^Postharvest Technology Unit, Department of Horticulture and Crop Production, University of Energy and Natural Resources, Sunyani, Ghana; ^3^School of Biosciences, University of Nottingham, Sutton Bonington Campus, Loughborough LE12 5RD, UK; ^4^Laboratory of Sustainable Agronomy and Crop Protection, Institute of Plantation Studies, Universiti Putra Malaysia, 43400 Serdang, Selangor, Malaysia

## Abstract

Several natural preservative techniques including plant extracts are used to minimize postharvest losses caused by pathogens. Our recent findings elucidated that the application of crude extracts of ginger, turmeric, and “dukung anak” (*Phyllanthus niruri* Linn.) alone causes phytotoxicity and adversely affects the postharvest quality of dragon fruit, especially at high concentrations. This study investigated the effect of a composite coating of 10% gum arabic (GA) and crude extracts of ginger, turmeric, and “dukung anak” separately at 5, 10, and 15 g L^−1^ on postharvest quality of dragon fruit stored at 11 ± 2°C, 80% RH for 28 days. After 28 days of cold storage, anthracnose was significantly reduced in fruit coated with 10% GA plus 10 or 15 g L^−1^ of any of the crude extracts and resolved the problem of phytotoxicity while maintaining the postharvest quality of fruit for 28 days. The reduction of anthracnose was pronounced at 10% GA+10 g L^−1^ of turmeric extract (38.6%) which was not significantly different at 10% GA+10 g L^−1^ of ginger extract compared to control (41.3%). Composite coating of 10% GA+10 g L^−1^ of turmeric extract maintained the postharvest quality of dragon fruit as was evident with a reduction in weight loss (2.53%), delayed degradation of titratable acids (0.15%), and maintained fruit firmness (28.72 N) and the overall acceptability of the fruit after 28 days. We conclude that incorporation of 10% GA with turmeric extract at a high concentration can serve as a potential biofungicide in postharvest management of fresh produced by reducing phytotoxicity while improving the overall acceptability of fruit.

## 1. Introduction

*Selenicereus costaricensis* (F.A.C. Weber) S. Arias and N. Korotkova (pitaya or red-fleshed pitaya, also known as *Hylocereus costaricensis*, and possibly known as *Hylocereus polyrhizus* (F.A.C. Weber) Britton and Rose) [1] is native to Colombia, Costa Rica, Nicaragua, Panamá, Peru, and Mexico and widely cultivated in tropical Southeast Asia regions such as Taiwan, Vietnam, Malaysia, and Central and South America [2]. The red-fleshed dragon fruit has unique bright-red or purple skin and prominent scales. The fruit is oval, elliptical, or pear-shaped, and the flesh has a sweet taste with edible interspersed tiny seeds [3]. It is rich in vitamins, beta-carotene, sugars, calcium, magnesium, and carbohydrates and is a good source of dietary fiber [4, 5]. The presence of betacyanin, a compound from a set of water-soluble nitrogen-containing pigments known as betalains, is responsible for the reddish color of the fruit, peel, and flesh [6], and betalains play a vital role as the major antioxidant contributor [6, 7]. Dragon fruit helps to protect cells from damage, cancer, and premature aging due to the presence of antioxidants, improves the balance of good and bad bacteria in the intestines, hence promoting the efficient digestion of food due to the presence of probiotics, and also helps to control blood sugar; therefore, it is recommended for diabetic patients [5].

However, its high perishability contributes to its short shelf life if proper postharvest management practices during transportation, storage, and marketing are not carried out. Generally, postharvest techniques employed in postharvest management of fruits such as dragon fruit require proper cold storage (low temperatures), which tends to minimize respiration rate and ripening and reduce early deterioration of fruit quality [8, 9]. However, one major biotic factor that affects dragon fruit quality during postharvest storage is anthracnose, a cosmopolitan disease caused by *Colletotrichum gloeosporioides* (Penz.) Penz. and Sacc. The disease is most common in tropical and subtropical fruits and can result in a 50% loss of the marketable yield [10].

Traditionally, some farmers in a bid to control postharvest diseases in fruits such as dragon fruit abuse the application of preharvest fungicides such as mancozeb by not following strictly the preharvest intervals. This results in an accumulation of chemical residues on fruits after harvest that could be detrimental to humans. Toxicological effects of the abuse and misuse of synthetic fungicides such as dithane M-45 (a mancozeb contact fungicide), dithiocarbamate, azoxystrobin, difenoconazole, cypermethrin, chlorpyrifos, difenoconazole, or/and *λ*-cyhalothrin, ethylene-bis-dithiocarbamate (EBDC), and propineb in tomato, dragon fruit, lettuce, rice, apple, and lettuce have been reported [11–16]. In most cases, producers (farmers), especially in developing countries, ignore the preharvest interval of these synthetic chemicals resulting in a significantly high residual effect on fresh produce during production. Even though mancozeb is practically not acutely toxic via the oral and dermal route of exposure, it is a mild skin irritant. However, chronic exposure leads to impaired thyroid function, birth defects, and cancer [13].

Residues of fungicides on fresh fruit are also potentially harmful to the environment and human health. Therefore, new and safer methods such as plant extracts and other natural products [8, 17–21] should be investigated and further developed into biofungicides in postharvest management of diseases in dragon fruit to reduce the use of synthetic fungicides.

Crude extracts of turmeric rhizome (*Curcuma longa* Linn.), *Phyllanthus niruri* Linn. (locally known in Malaysia as “Dokung or dukung anak”), and ginger (*Zingiber officinale* Roscoe) are rich in bioactive compounds with vast antifungal activity against phytopathogens including *C. gloeosporioides* and other clinical pathogens [22–27]. Volatile oils, containing turmerone, and other coloring agents called curcuminoids are major components of turmeric [28]. These curcuminoids (curcumin, desmethoxycurcumin, and bisdemethoxycurcumin) are compounds responsible for the yellow color and also rich in antioxidants where curcumin forms the major bioactive component of turmeric [29, 30]. The antimicrobial effect of *Phyllanthus* spp. against several phytopathogens such as *Colletotrichum* spp. is due to the presence of two major alkaloids allosecurinine and securinine [26, 31, 32]. Gingerols and shogaol are known bioactive compounds responsible for many antifungal activities of ginger against several clinical and phytopathogens [27, 33]. These plants are generally regarded as safe (GRAS), nontoxic to humans, biodegradable, cheap, and do not leave residual effects on fruits, making them a good postharvest agent in managing diseases of dragon fruit.

Several edible coating films from polysaccharides have been reported for fruit postharvest preservation. For instance, edible coating formulations based on hydroxypropyl methylcellulose [34], wax coating [35], sodium alginate [36], Persian gum [37], carnauba wax [38], pea starch and guar gum [39], aloe vera gel [40], methylcellulose, carboxymethyl cellulose (CMC), and chitosan [41] maintained fruit quality and extended postharvest storage-life of fruits. Among the potential edible coatings, gum arabic (GA), a natural water-soluble polysaccharide, could be used as an edible coating for the postharvest preservation of horticultural fruits. GA is derived from the gum exudates of *Acacia senegal* tree and used as a natural film preservative due to its water solubility, film forming, antioxidant activity, and emulsification properties and is generally regarded as safe (GRAS) [21, 42]. Studies have proven that GA coating either alone or incorporated with other preservative agents reduced postharvest decay and maintained the overall postharvest quality of harvested fruits such as banana, papaya, mango, tomato, ponkan fruits, and guava fruits [18, 21, 43–45]. The combination of GA incorporated with cinnamon essentially reduced anthracnose in banana and papaya after 28 days followed by 5 days of shelf life at room temperature [46]. Other studies reported a 70%-80% reduction of anthracnose in banana fruits after treatment with 10% GA+0.75% or 1% chitosan after 28 days of storage at 13°C [19, 20]. Additionally, the incorporation of 10% GA with 3% calcium chloride or only calcium chloride significantly controlled anthracnose in mango and dragon fruit [47, 48]. GA has no antifungal effect but plays a significant physicochemical role in maintaining fruit quality postharvest [19, 20]. Additionally, GA is known to reduce phytotoxicity in some natural products while maintaining the postharvest quality of fruits [46].

A recent study shows that the use of plant extracts at high concentrations is detrimental to the postharvest quality of dragon fruit. Concentrations of 5 g L^−1^ and above (10 or 15 g L^−1^) of ginger crude extract, 10 or 15 g L^−1^ of turmeric extract, and 15 g L^−1^ of dukung anak cause phytotoxicity, thereby compounding disease incidence in dragon fruit after 28 days of cold storage [22]. Therefore, this study investigated for the first time the efficacy of ginger, turmeric, and dukung anak extracts incorporated with gum arabic on postharvest management of dragon fruits aiming at minimizing phytotoxicity, reducing anthracnose caused by *Colletotrichum gloeosporioides* as well as improving the physicochemical qualities of dragon fruit during cold storage.

## 2. Material and Methods

### 2.1. Dragon Fruits

In this experiment, disease-free, medium, and uniform *Hylocereus costaricensis* var. HU1 (Pink Dragon Sunlike) (among the two registered varieties under the Malaysia national listing) at matured stage (purplish pink color) of weight 420-480 g (grade A) were purchased from a commercial farm called Golden River farm located at Sg. Sompo in Lenggeng, Malaysia, during the harvest season (September). The fruits were washed with clean water to get rid of debris and soil and then disinfected with 1% sodium hypochlorite. Fruits were washed with distilled water after disinfection and air-dried at room temperature.

### 2.2. Treatment Materials

Plant crude extracts turmeric *(Curcuma longa* Linn.), ginger (*Zingiber officinale* Roscoe cv. “Bentong”), and Dukung Anak” powder (*Phyllanthus niruri*) at different concentrations incorporated separately with gum arabic (GA) served as treatments. GA powder (500 g) CP grade was purchased from Sigma-Aldrich. Disease-free turmeric rhizomes (*Curcuma longa* Linn.) and ginger (*Zingiber officinale* Roscoe cv. “Bentong”) were purchased from commercial farms Bentong (along the Titiwangsa mountain) and a commercial supplier (at Farm Price SDN.BHD in Johor), respectively. “Dukung Anak” powder (*Phyllanthus niruri*) was purchased from Ethnoherb Resources, Malaysia. Ginger and turmeric rhizomes were disinfected with 1% sodium hypochlorite, followed by washing with distilled water before oven-drying or drying at room temperature and crude extraction.

### 2.3. Crude Extraction of Plants

Crude extraction was carried out according to Bordoh et al. [22]. The disinfected and washed tissues of turmeric and ginger were sliced into pieces, sun-dried for a week, and oven-dried at 50°C in a hot air oven for 6 h to achieve a moisture content of 11.54 ± 12%. Each dried rhizome was pulverized to a coarse powder (0.5 mm) using an ultracentrifugal mill ZM 200 before extraction. Powdered samples of 200-250 g were soaked in a 1.0 L conical flask containing 0.8 L of methanol for 24 h. Extractions (5-6x) were done, and the total extract solution was pooled and stored at room temperature in a 1.0 L conical flask. Extract solutions obtained were filtered through a muslin cloth to remove impurities before rotatory evaporation. The collected solution was concentrated and dried under reduced pressure on a rotary evaporator in 40°C water bath to obtain the crude extracts.

### 2.4. Formulation of Composite Coating of Gum Arabic and Plant Extracts

#### 2.4.1. Preparation of Stock Gum Arabic

GA (GA powder, 0.5 kg, CP grade, Sigma-Aldrich) solution was prepared according to Maqbool et al. [19]. About 0.15 kg of gum arabic powder was dissolved in 1.5 L of ULTRA pure water (PURELAB ELBA system Option-R&BP; Veolia Water Systems, High Wycombe, UK) to obtain 10% GA. The GA solution was stirred constantly at low heat (40°C) using a hot plate magnetic stirrer (Model LHS-HTS-1003; Bunkyo-Ku, Tokyo, Japan) for 60 min until a brownish color is obtained. The solution was then filtered through four layers of cheesecloth to remove any undissolved impurities.

#### 2.4.2. Incorporation of Gum Arabic with Plant Extracts

A stock solution of the formulation was prepared according to Jaapar et al. [49], with slight modifications. A known weight of each crude extract was redissolved with a 0.05 mol fraction of methanol, and the final aqueous stock solution for each plant extract was made to 10% GA+22.17 g L^−1^ by addition of 10% GA solution (*v*/*v*). A working concentration (treatment) compromised of 10% GA+5 g L^−1^, 10% GA+10 g L^−1^, and 10% GA+15 g L^−1^ per plant extract was prepared by diluting the stock with already prepared 10% GA solution. The pH of each working solution was adjusted to 5.6 by adding 1.0 M NaOH using a digital pH meter (Model CyberScan pH 510; Eutech Instruments Pte. Ltd. Singapore) while ensuring there is no change in the concentration of the treatments. Uniformity of the layer of composite coating is attained when the aqueous brownish color of GA changes to slight green, deep yellow, and pale yellow after the addition of *Phyllanthus niruri*, turmeric, and ginger extract, respectively.

### 2.5. Effect of Composite Coating on Disease Incidence and Severity

#### 2.5.1. Application of Formulation on Fruits

Matured dragon fruit (purplish-pink color) of uniform size and with no deformity were washed with sodium hypochlorite (1%), rinsed with purified water, and air-dried at room temperature (25 ± 2°C). The fruit was then inoculated, dipped for 2 min in a spore suspension of *C. gloeosporioides* (10^5^ spores/mL), and air-dried completely at ambient (25 ± 2°C). Inoculated air-dried fruit were dipped for 2 min in each treatment and dried at room temperature. Inoculated fruit alone and those treated with a fungicide (mancozeb-2 g L^−1^) for 1 min served as the negative and positive control, respectively. Previous findings from our *in vitro* studies showed that at 0.05 mol fraction of methanol (cosolvent), there was no antifungal activity against *C. gloeosporioides* since all methanol had evaporated at room temperature [22]; therefore, methanol-treated fruit was not added as a control. The fruit was air-dried at room temperature before packing in commercial packaging cartons and stored at 11 ± 2°C, 80% RH for 28 days in a complete randomized design.

#### 2.5.2. Disease Incidence and Disease Severity

The effect of formulation on disease incidence and disease severity was evaluated weekly for 28 days during cold storage. Disease incidence data were presented as the percentage of fruit showing anthracnose out of the total number of fruits in each treatment, while disease severity was scored following the scale (1: 0% of fruit surface rotten; 2: 1–25%; 3: 26–50%; 4: 51–75%; and 5: 76–100% rotten) [50]. A total of 330 fruits were used. For each formulation, a total of 90 fruits were used. The experiment was done in triplicate.

#### 2.5.3. Evaluation of External Quality of Fruit after Formulation Application

An observational study was conducted with 20 participants to assess the external appearance of fruit after coating [22]. The evaluation focused mainly on consumer appeal about fruit peel appearance and smell, thus which formulation (treatments) either improved or compromised the original color and smell of dragon fruit. The evaluation was carried out after 21 days of cold storage when the fruit was still edible.

### 2.6. Efficacy of Formulation on Postharvest Quality

#### 2.6.1. Physicochemical Quality of Dragon Fruit

For physical quality analysis (weight loss, color, and fruit firmness), a total of 15 fruits were used for each treatment. The experiment was done in triplicate. For the chemical quality analysis, i.e., total soluble solids (TSS) and titratable acidity (TA), a total of 5 fruits were randomly sampled. The physiochemical quality analysis was conducted every week for a period of 21 or 28 days depending on the parameter.

*(1) Weight Loss*. Weight was measured using a digital balance (Model GF-6100, A&D Co. Ltd., Japan) weekly for 28 days and expressed as a percentage on a fresh weight basis.

*(2) Peel Color Change*. Peel color was analyzed using the Hunter Lab System, Miniscan XE Plus colorimeter (model: 45/0-5, Reston Virginia, USA). The colorimeter was equipped with a measuring head that had an 8.0 mm diameter measuring area and was calibrated with standard black and white tiles. The Miniscan XE Plus colorimeter was calibrated using black and white tiles with values of *X* = 79.0, *Y* = 83.9, and *Z* = 87.9. The peel color determination was expressed in chromaticity values of *h*°. The *h*° (hue angle) = angle of tangent − 1 *b*∗/*a*∗, where 0 = red-purple, 90 = yellow, 180° = blue-green, and 270° = blue [46].

*(3) Fruit Firmness*. Instron Universal Testing Machine with a 5.0 mm plunger tip, single-column model (Instron 2519-104, Norwood, MA) interfaced with a computer was used to determine the fruit firmness, by measuring the amount of force (N) to puncture a hole in the fruit on each sampling day. The machine was set for maximum compression with a speed of 20.0 mm/min [46].

*(4) Total Soluble Solids*. TSS was determined according to Dávila-Aviña et al. [51] with slight modification with a total of 5 fruit. Fruit pulp (10.0 g) was frozen for 24 hr and homogenized using a kitchen blender with 40 mL of purified distilled water. The solution was centrifuged at 5000 x g for 10 min using Eppendorf centrifuge 5810 R (Eppendorf AG, Hamburg). The obtained filtrate (fruit juice) was used for TSS and TA analysis. TSS (%) was determined using a Palette Digital Refractometer (model: PR-32*α* Atago Co., Ltd. Japan). An aliquot of 20.0 *μ*L of filtrate (fruit juice) was placed on the prism glass of the refractometer, which has already been calibrated with distilled water.

*(5) Titratable Acidity*. The titratable acidity (TA) of fruit was performed and calculated using the titration method of Ranganna [52]. An aliquot of 5.0 mL of fruit juice obtained for the analysis of TSS was placed in a beaker, and two drops of 0.1% phenolphthalein (R & M Chemicals, UK) were added as an indicator and titrated against 0.1 N NaOH (Merck, Germany) until an endpoint color of pink (pH 8.1). The result was expressed as the percentage of citric acid.

#### 2.6.2. Effect of Formulation on Respiration Rate and Ethylene Production

*(1) Respiration Rate*. Respiration rate as indicated by CO_2_ production was measured according to Maqbool et al. [46] with slight modification. Three medium-sized dragon fruits of approximate weight 0.54 kg were placed in a 2.0 L plastic container for 2 h, and 1.0 mL of gas sample was withdrawn from the headspace with a gastight hypodermic syringe and analyzed with a gas chromatograph (GC) (Clarus-500, Perkin-Elmer, USA) equipped with a stainless-steel column (Porapak R 80/100). Helium served as the carrier gas at a flow rate of 20.0 mL/min. Temperatures were 60, 100, and 200°C for the oven, injector, and thermal conductivity detector (TCD), respectively. One milliliter of CO_2_ gas (1.0%) (Scotty Gases, Bellefonte, PA, UK) was used as the external standard for calibration. The experiment was done in triplicate per treatment, and the time point for measurement was weekly until the end of the storage period. The amount of CO_2_ produced was expressed in mL CO_2_ kg^−1^·h^−1^.

*(2) Ethylene Production*. Ethylene evolution was measured by taking a 1.0 mL gas sample produced from the same number of fruits from each jar as described for respiration rate using a hypodermic syringe and injecting it into a GC. The GC was equipped with a stainless-steel column (Porapak T, 100/120) and a flame ionization detector (FID). Nitrogen, hydrogen, and air flow rates were 20.0 mL/min. Nitrogen served as a carrier gas. Temperatures were 150, 200, and 200°C for the oven, injector, and FID, respectively. One milliliter of 10.0 *μ*L·mL^−1^ pure ethylene gas (Scotty Gases, Bellefonte, PA, UK) was used as an external gas standard for calibration. The amount of ethylene was expressed in *μ*L C_2_H_4_ kg^−1^·h^−1^.

### 2.7. Effect of Formulation on Sensory Evaluation

Sensory evaluation of pulp (fruit flesh), pulp color, texture, aroma, and overall acceptability for all treatments was performed at the end of 21 days of storage using the method of Bai et al. [53] with slight modification. The best composite coatings that significantly controlled disease incidence and severity and maintained the physicochemical quality of dragon fruit were used. The control consists of fruit washed with only distilled water. A total of forty-two (42) fruits were used for sensory evaluation. For each treatment, six fruits were randomly selected for this analysis. Panelists were asked to score the difference among all the samples by allotting numbers on appearance, pulp color, aroma, sweetness, texture, and overall acceptability. Each panelist was asked to rate each parameter based on the rating as follows: 1: extreme dislike; 2: dislike; 3: acceptable; 4: good; and 5: excellent.

## 3. Data Analysis

Two-way ANOVA was performed for the efficacy of extracts on DI and DS and postharvest quality of fruits except for sensory evaluation where one-way ANOVA was performed. All analysis was performed using the computer software Genstat version 18^th^ edition (VSNI product). Means were separated using Fisher's unprotected test at (*P* < 0.05). Disease incidence data were transformed (arcsine of the square root of the proportion of affected fruit) before analysis. Experiments were repeated twice, and data were pooled.

## 4. Results

### 4.1. Efficacy of Formulation on Disease Incidence and External Quality Appearance

#### 4.1.1. Disease Incidence

**Figure 1:**
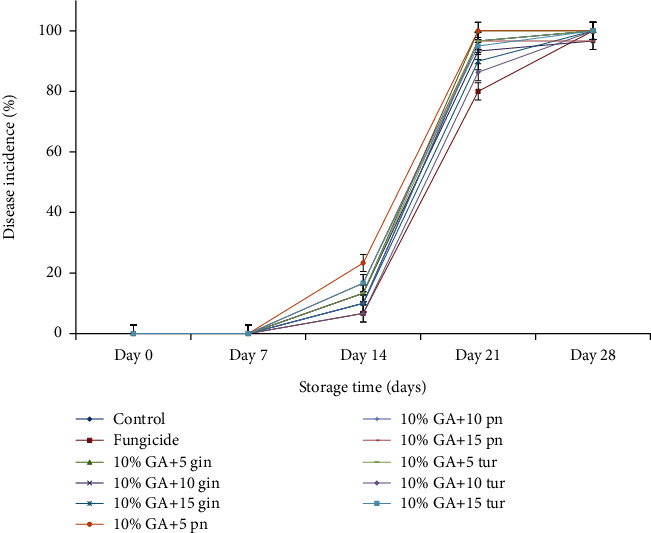
Effect of 10% GA incorporated with plant extracts at different concentrations on disease incidence during cold storage for 28 days. 10% GA+5 gin, 10% GA+10 gin, and 10% GA+15 gin mean 10% gum arabic incorporated with 5, 10, and 15 g L^−1^ of ginger extracts. tur: turmeric crude extract; pn: dukung anak crude extract. The explanation for ginger is similar to dukung anak and turmeric extract.

DI increased progressively with storage time in both control and treated fruit (Figure 1). DI started after day 14 and increased until day 28 by which time all treated and control fruit recorded 100% DI (Figure 1). It is imperative to state that the efficacy of the formulation to reduce DI in dragon fruit was best on day 21, since on day 28, all fruit showed severe symptoms of anthracnose and other secondary infections.

**Figure 2:**
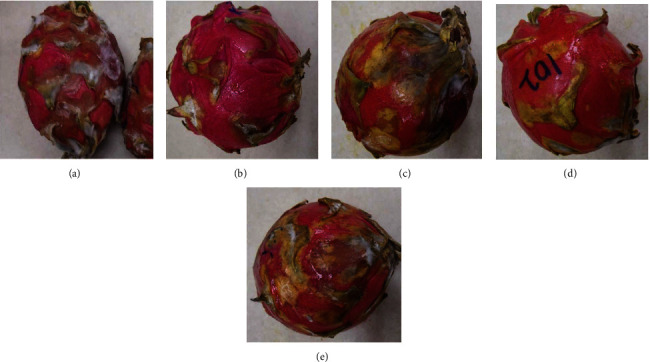
Effect of 10% GA incorporated with turmeric extract at different concentrations (g L^−1^) on disease incidence and severity of dragon fruit after 28 days of storage. (a) Control, (b) fungicide (mancozeb-2 g L^−1^), (c) 10% GA+5 g L^−1^, (d) 10% GA+10 g L^−1^, and (e) 10% GA +15 g L^−1^.

**Figure 3:**
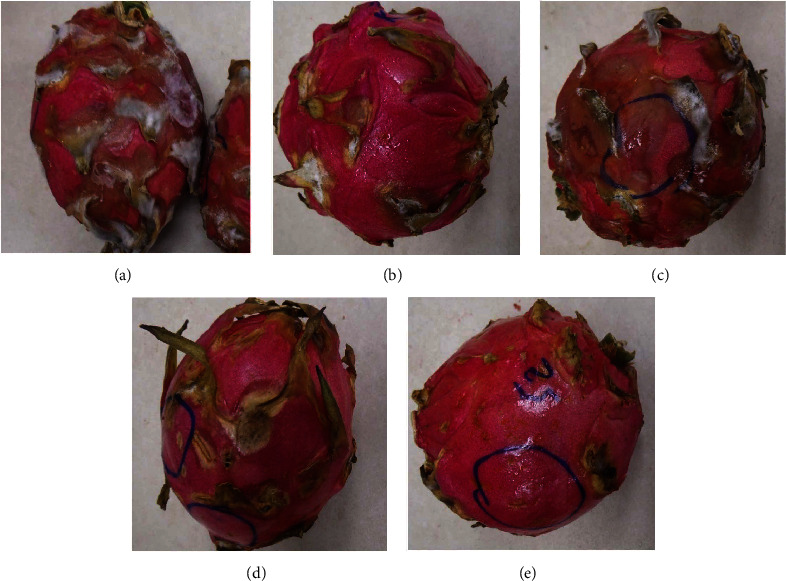
Effect of 10% GA incorporated with ginger extract at different concentrations on disease incidence and severity of dragon fruits after 28 days of cold storage. (a) Control, (b) fungicide (mancozeb-2 g L^−1^), (c) 10% GA+5 g L^−1^, (d) 10% GA+10 g L^−1^, and (e) 10% GA +15 g L^−1^.

On day 21, fruit treated with 10% GA plus 10 or 15 g L^−1^ irrespective of the plant extracts markedly recorded low DI even though it was not different from the control compared to the fungicide mancozeb which recorded the lowest (*P* < 0.05) DI. At the end of 28 days of storage, fruit treated with 10% GA+10 g L^−1^ of turmeric recorded (*P* < 0.05) a low DI comparable to mancozeb (Figure 2) which was not different from fruit treated with 10% GA plus either 10 g L^−1^ or 15 g L^−1^ of ginger extract compared to control (Figure 3). Fruit treated with 10% GA plus dukung anak extract did not show any significance in reducing anthracnose irrespective of the concentration used compared to the control (Figure 1).

#### 4.1.2. Disease Severity

**Figure 4:**
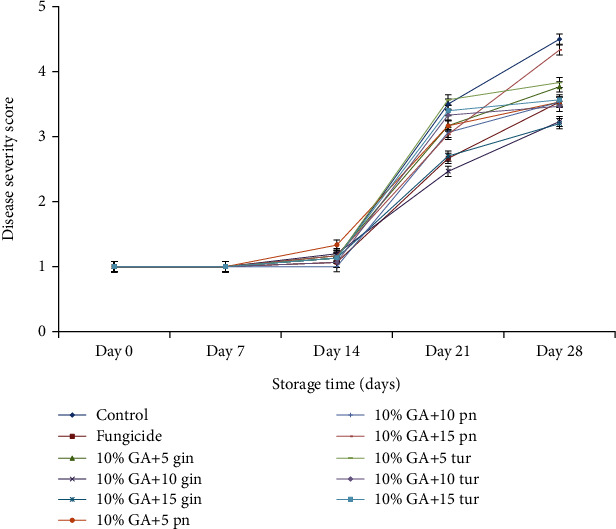
Effect of 10% GA incorporated with plant extracts at different concentrations on disease severity during cold storage for 28 days.

Like DI, disease severity progressed significantly (*P* < 0.05) with storage time, and it was low in treated fruit compared to the control (Figure 4). A DS score-1 (an indication of no DS) was observed from day 7 until after day 14, which marked the onset of the disease. On day 21, fruit treated with 10% GA+10 g L^−1^ of the ginger extract significantly recorded a low DS (DS score-2.45) compared to the control (DS score-3.5).

At the end of the 28 days of storage, DS was significantly (*P* < 0.05) low in fruit treated with 10% GA+10 g L^−1^ of ginger extract (DS score-3.23) comparable to mancozeb, and it was not different in 10% GA+10 g L^−1^ of turmeric extract fruit (DS score-3.26) compared to control (DS score-4.5) (Figure 4). Fruit treated with 10% GA+5 g L^−1^ or 15 g L^−1^ of either dukung anak or turmeric did not significantly reduce DS.

### 4.2. External Appearance of Fruit after Formulation Application

**Figure 5:**
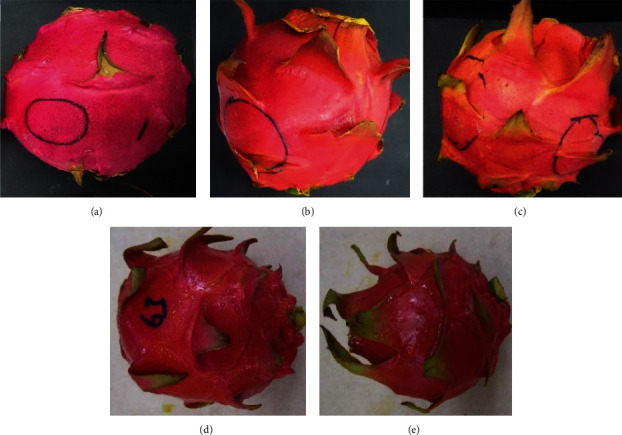
The external quality appearance of turmeric-coated fruit alone (upper row) and after incorporation with 10% GA (lower row) after 21 days of storage. Upper row (a–c): (a) control, (b) fruit coated with only 15 g L^−1^ of turmeric, (c) fruit coated with only 10 g L^−1^ of turmeric. Lower row (d, e): (d) fruit coated with 10% GA+15 g L^−1^ of turmeric, (e) 10% GA+ 10 g L^−1^ of turmeric.

The incorporation of 10% GA with plant extracts markedly enhanced the external appearance of the fruit, even at high concentrations. All treated fruit, irrespective of the plant extract, had an enhanced external glossy appearance after incorporation with 10% GA (Figure 5; lower row, 10% GA+15 g L^−1^ and 10% GA+10 g L^−1^). Additionally, the dragon fruit's original color was maintained when 10% GA was incorporated with crude extract, especially in turmeric or ginger-treated fruit (Figure 5; lower row, 10% GA+15 g L^−1^ and 10% GA+10 g L^−1^) compared to when fruits were coated with only extract (Figure 5; upper row 15 g L^−1^ and 10 g L^−1^).

Incorporation of 10% GA with plant extracts to some extent reduced the intensity of crude extract smell, especially in ginger and turmeric-coated fruit at 10 g L^−1^ or 15 g L^−1^, allowing the dragon fruit to maintain its original smell and color.

### 4.3. Efficacy of Extract on Physicochemical Quality of Fruit

#### 4.3.1. Weight Loss

**Figure 6:**
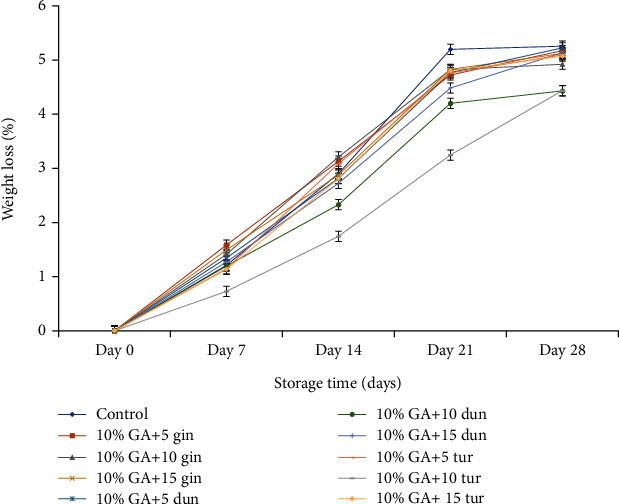
Effect of 10% GA incorporated with plant extracts at different concentrations on weight loss (%) during cold storage for 28 days.

Weight loss increased progressively (*P* < 0.05) with storage time in both treated and control fruit, and it was high in control after 28 days (Figure 6). On day 21 (recommended edible stage for this study), weight loss was significantly (*P* < 0.05) low in fruit coated with 10% GA+10 g L^−1^ of turmeric (3.24%) compared to control (5.19%).

At the end of the 28 days of storage, fruits treated with 10% GA+10 g L^−1^ of turmeric crude extract recorded low weight loss followed closely by dukung anak-coated fruits at 10% GA+10 g L^−1^ compared to control fruits.

#### 4.3.2. Firmness

**Figure 7:**
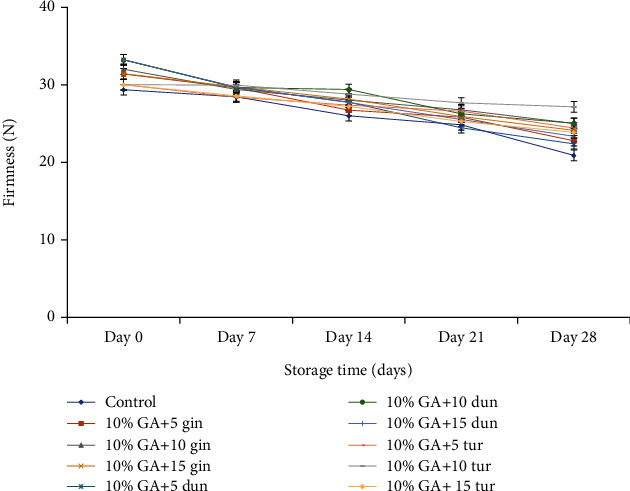
Effect of 10% GA incorporated with plant extracts at different concentrations on firmness (N) during cold storage for 28 days.

Like weight loss, firmness decreased (*P* < 0.05) with storage time in both treated and control fruit after storage (Figure 7). Despite a decline in fruit firmness, firmness was relatively high in treated fruit compared to control fruit.

It was evident that the composite coating of 10% GA+10 g L^−1^ of any crude extract (either turmeric or ginger or dukung anak) significantly (*P* < 0.05) helped to maintain fruit firmness. However, the fruit was firmer especially at 10% GA+10 g L^−1^ of ginger extract, which was not significantly different from turmeric-coated fruit at the same concentrations (10% GA+10 g L^−1^) compared to the control after 28 days of storage.

#### 4.3.3. Fruit Peel Color Development

Color development was measured on day 21 and not on day 28, due to severe disease incidence on day 28 which rendered peel tissue unmeasurable with the colorimeter.

**Figure 8:**
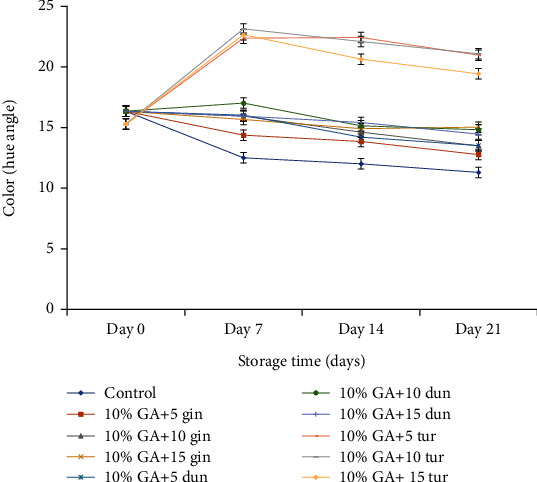
Effect of 10% GA incorporated with plant extracts at different concentrations on color development during cold storage for 21 days.

Low *h*° indicates fruit senescence and ripening, and this was evident as dragon fruit peel had a dark color due to the development of reddish color in the peel as the pigmentation process occurred during the postharvest storage [54]. Color development was delayed (*P* < 0.05) in fruit treated with 10% GA plus turmeric crude extract at any concentration, but this was pronounced at 10% GA+10 g L^−1^ of turmeric extract compared to the control (Figure 8).

#### 4.3.4. Total Soluble Solids and Titratable Acidity

**Figure 9:**
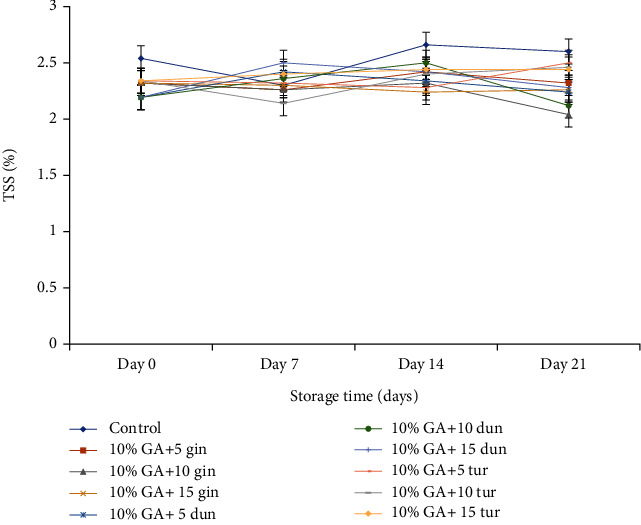
Effect of 10% GA incorporated with plant extracts at different concentrations on TSS during cold storage for 21 days.

TSS and TA were determined on day 21 since most of the fruit was not edible on day 28 due to severe DI. TSS increased steadily (*P* < 0.05) but was maintained in treated fruit. On day 21, TSS was significantly (*P* < 0.05) low at 10% GA+10 g L^−1^ of ginger-coated fruit (2.04%), which was not different in the composite coating of dukung anak-coated fruit at 10% GA+10 g L^−1^ (2.12%) or turmeric-coated fruit at 10% GA+10 g L^−1^ (2.11%) compared to control (2.60%) (Figure 9).

**Figure 10:**
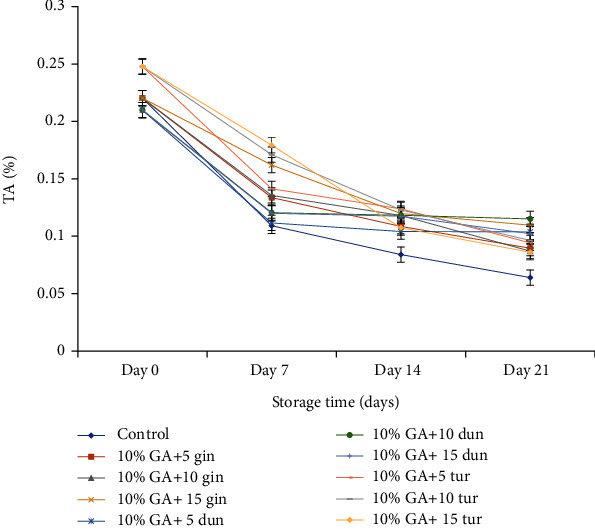
Effect of 10% GA incorporated with plant extracts at different concentrations on TA during cold storage for 21 days.

TA decreased steadily (*P* < 0.05) with storage time in both treated and control fruit. Unlike TSS, TA was relatively high in treated fruit compared to control (Figure 10). At the end of 21 days of cold storage, TA was high in fruit treated with 10% GA+10 g L^−1^ of turmeric extract, which was not different in fruit treated with 10% GA+15 g L^−1^ of ginger or 10% GA+10 g L^−1^ of dukung anak-coated fruit compared to control (Figure 10).

### 4.4. Efficacy of Formulation on Respiration Rate and Ethylene Production

**Figure 11:**
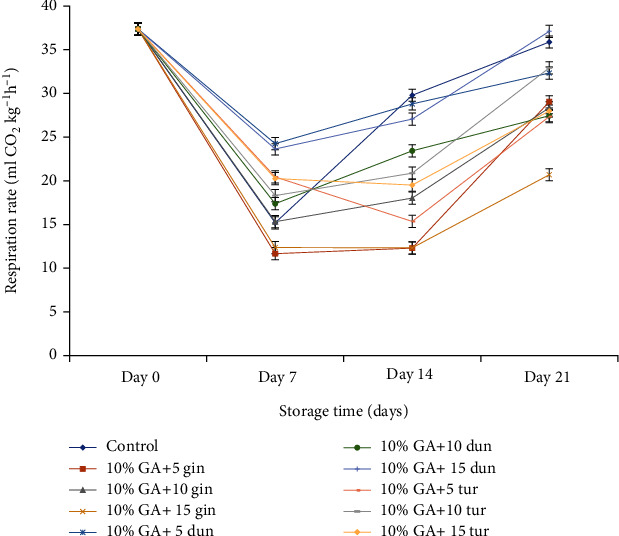
Effect of 10% GA incorporated with plant extracts at different concentrations on respiration rate during cold storage for 21 days.

A continuous decline in the respiration rate occurred in all treated fruit compared to the control. A sudden decrease in respiration rate was observed on the 7^th^ day and was maintained until day 14 but increased afterward until day 21 (Figure 11). On day 21, respiration rate was significantly low (*P* < 0.05) when fruit was treated with 10% GA+15 g L^−1^ of ginger crude extract (20.69 mL CO_2_ kg^−1^·h^−1^), followed closely by fruit coated with 10% GA+5 g·L^−1^ of turmeric extract (27.35 mL CO_2_ kg^−1^·h^−1^) which was not different from fruit coated with 10% GA+10 g·L^−1^ ginger extract compared to control (35.88 mL CO_2_ kg^−1^·h^−1^).

**Figure 12:**
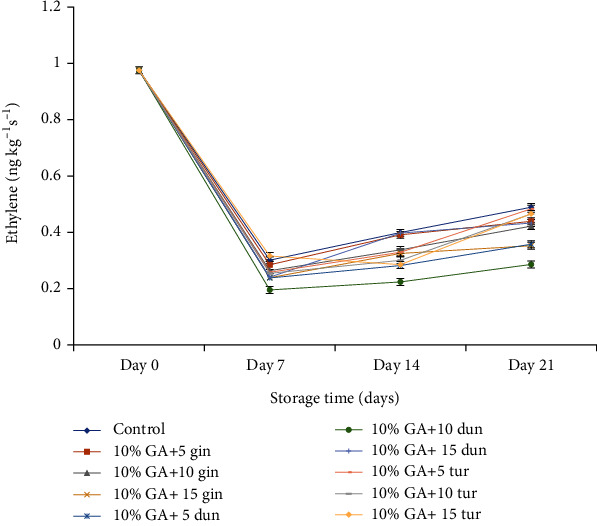
Effect of 10% GA incorporated with plant extracts at different concentrations on ethylene production during cold storage for 21 days.

At the end of 21 days of storage, the respiration rate was significantly low (*P* < 0.05) in fruit coated with 10% GA+10 or 15 g L^−1^ of ginger extract compared to control. Ethylene production decreased with storage time with a transient increase from day 7 until the end of 21 days of storage (Figure 12), contrary to respiration rate which increased drastically after day 14 until the end of 21 d storage.

Ethylene production was significantly low in fruit treated with 10% GA+10 g·L^−1^ of dukung anak crude extract (0.28 *μ*L C_2_H_4_ kg^−1^·h^−1^), followed closely by fruit treated with 10% GA+10 g·L^−1^ ginger extract 0.35 *μ*L C_2_H_4_ kg^−1^·h^−1^ compared to control (0.48 *μ*L C_2_H_4_ kg^−1^·h^−1^) at day 21 (Figure 12). After 21 days of cold storage, ethylene production was significantly low (*P* < 0.05) in fruit treated with 10% GA+10 g·L^−1^ of dukung anak, which was not different in fruit treated with 10% GA+10 g·L^−1^ of ginger crude extract compared to the control.

### 4.5. Sensory Evaluation

Sensory evaluation of treated and control fruit at the end of 21 days of storage revealed significant (*P* < 0.05) differences in appearance, pulp color, aroma, sweetness, texture, and overall acceptability.

There was a varied opinion by panelists on all parameters during sensory evaluation. However, fruit coated with 10% GA+10 g L^−1^ of turmeric attained the highest score in pulp color, aroma, sweetness, texture, and overall acceptability during the sensory evaluation (Table 1). This shows that fruit coated with 10% GA+10 g L^−1^ of turmeric was widely accepted and good to the panelist compared to the control.

## 5. Discussion

### 5.1. Efficacy of Formulation on Disease Incidence and Severity

While GA has extensively been used as a hydrocolloid in the food industry [55, 56], no study on its antifungal or fungitoxicity has been reported [19]. In this present study, there was a synergistic effect of composite coatings of 10% GA+10 g L^−1^ of ginger or turmeric extract that delayed anthracnose and its severity. The 10% GA served as a carrier for the 10 g L^−1^ crude extract, allowing the slow release of bioactive compounds in the crude extract to control anthracnose during postharvest storage. Bioactive compounds such as gingerols and curcumin [23, 57–60] in 10 g L^−1^ of ginger and turmeric, respectively, helped to control anthracnose and its severity. Our study confirms the findings from Maqbool et al. [19]; Maqbool et al. [20]; Maqbool et al. [46], and Khaliq et al. [48], where natural products such as chitosan, essential oils incorporated with GA synergistically helped to control anthracnose in banana, papaya, and tomatoes while extending their shelf life. On the contrary, this study contradicts research by Bordoh et al. [22] who stated that a high concentration of 10 or 15 g L^−1^ ginger or turmeric extract resulted in phytotoxicity thereby compounding disease incidence and severity. In this study, the incorporation of 10% GA with a high concentration (10 or 15 g L^−1^) of ginger or turmeric extract significantly reduced disease incidence and severity without any phytotoxicity. This is because 10% GA to some extent reduced the phytotoxicity by forming a semipermeable membrane or a coating, thereby preventing the direct contact of extract to the fruit peel, hence reducing tissue damage.

### 5.2. Efficacy of Formulation on Physicochemical Quality

#### 5.2.1. Weight Loss

The thickness of the fruit skin determines its susceptibility to rapid water loss resulting in shriveling and deterioration [61]. Hence, using edible coating can maintain the quality of fruit by maintaining the turgidity through water retention in the fruit [62]. The advent of DI generates peel tissue damage which increases respiration, resulting in excessive weight loss. In the present study, the reduction in weight loss, especially in fruit coated with 10% GA+10 g L^−1^ of turmeric was due to the synergistic effect of the composite coating where the 10% GA served as a semipermeable barrier against oxygen, carbon dioxide, and moisture, thus reducing respiration, water loss, and oxidation reactions [63, 64], while 10 g L^−1^ was fungistatic against the fungus, hence reducing disease incidence. Composite coating of GA combined with other natural products (cinnamon oil and propolis) or synthetic chemicals (calcium chloride) or silver nanoparticles delayed senescence by decreasing weight loss in banana, mango, green bell pepper, and papaya [19, 20, 46, 48, 65].

On the contrary, composite coating of 10% GA plus either 5 g L^−1^ or 15 g L^−1^ crude extract (especially ginger) recorded significantly high weight loss, which could be due to the thickness of the composite coating. At 10% GA+5 g L^−1^ of ginger crude extract, the composite coating was not thick enough to provide a significant barrier against water loss [46]. The high weight loss in fruit coated with 10% GA+15 g L^−1^ of the crude extract could be due to excessive heat generation due to 10% GA and the high amount of lipids (oils) in ginger at 15 g L^−1^ that led to the increase in anaerobic respiration since the composite coating was thick and completely blocked the lenticels [46]. Similarly, Zahid et al. [9] reported that the high weight loss of dragon fruit was due to the heat production of fruit coated with a high concentration of ethanolic propolis. Ghasemnezhad et al. [66] also reported high weight loss when a high concentration of chitosan was applied as an edible coating in apricot. Maqbool et al. [46] also reported high weight loss in the composite coating of 20% GA plus 1.0% CH in bananas and concluded that composite coating on banana fruit might be due to the generation of heat and production of end products from anaerobic fermentation.

#### 5.2.2. Firmness

In this study, the synergistic effect of 10% GA+10 g L^−1^ of turmeric extract enhanced fruit firmness. We propose that the 10 g L^−1^ of turmeric extract inhibited the fungal attack on the fruit peel, reducing tissue damage, that led to enhancing fruit firmness, while 10% GA provided a film-forming membrane responsible for delaying ripening, which could be due to low levels of oxygen and relatively high levels of carbon dioxide restricting the activities of cell wall degrading enzymes thereby retaining fruit firmness during storage. Our findings confirm similar research, where a composite coating of chitosan and GA, ethanolic extract of propolis and GA, and application of ethanol extract of propolis resulted in a modified atmosphere effect, thereby reducing moisture loss and retention of fruit firmness in banana, papaya, and dragon fruit [9, 46]. Fruit coated with 10% GA+5 g L^−1^ irrespective of plant crude extract could not maintain fruit firmness, and this was probably due to the low antifungal interaction between the host-pathogen at 5 g L^−1^, thereby leading to disease infection due to tissue damage by the fungus, which in turn increased weight loss and decreased firmness, while at 10% GA+15 g L^−1^, the decrease in firmness could be due to anaerobic respiration leading to the generation of heat and production of end products, since this composite coating was too thick. This confirms a study by Maqbool et al. [46] where banana fruit lost its firmness when the fruit was coated with 20% GA plus 1.0% CH.

#### 5.2.3. Peel Color Change

Fruit coated with 10% GA+10 g L^−1^ of turmeric extract had low reddish to dark color saturation. This could be attributed to a reduced respiration rate and ethylene production due to the composite effect that modified the gas exchange of fruit. This phenomenon helped to retard the ripening and senescence, ultimately retarding color change due to changes in peel chlorophyll content during fruit development. Nerd and Mizrahi [67] reported that the change of peel color to pale green and then to reddish of pitaya fruit was due to changes in peel chlorophyll content during fruit development. Our study confirms the research by Hedayati and Niakousari [65] where 10% GA combined with silver nanoparticles as a composite coating significantly delayed color changes in green bell pepper for 21 days. Similarly, Ali et al. [17] also reported that composite coating of ethanolic extract of propolis (1.5%) and 10% GA reduced the occurrence of anthracnose and delayed color changes in papaya.

#### 5.2.4. Total Soluble Solids and Titratable Acidity

An increase in respiration results in a corresponding increase in ripening leading to a sharp rise in TSS due to the breakdown of carbohydrates into sugar [47, 68]. The reduction of TSS is associated with the utilization of sugars as carbon skeleton sources for the fungus [47]. Therefore, a suppressed respiration rate will slow down the synthesis and use of metabolites, resulting in lower soluble solids contents due to the slow hydrolysis of carbohydrates to sugar [69, 70]. In this study, TSS was low in ginger-coated fruit at 10% GA+10 g L^−1^ which was not significantly different in turmeric-coated fruit at 10% GA+10 g L^−1^ or dukung anak-coated fruit at 10% GA+10 g L^−1^ compared to control. The low TSS at 10% GA+10 g L ^−1^ of all extracts was due to the synergistic effect of 10% GA that modified the internal gas exchange of the fruit, thereby reducing respiration and ethylene production [71] contributing to slow hydrolysis of carbohydrates to sugars [70]. The 10 g L^−1^ of the extract (ginger or turmeric) was the optimum concentration that inhibited *C gloeosporioides* utilization of sugars in the dragon fruit as their nutrient source for growth [72, 73]. Kamilova et al. [74] reported a significant reduction in sugars and acids in infected tomatoes due to fungal infection. Wang and Galletta [75] confirmed this when anthracnose-infected fruit recorded low soluble solid concentration and TA than healthy fruit.

Citric acid is the major organic acid in matured ripe dragon fruit [76]. TA was high in turmeric-coated fruit at 10% GA+10 g L^−1^, and this was not different in fruit coated with 10% GA+10 g L^−1^ of ginger. The decline in TA during postharvest storage is because organic acids, which serve as respiratory substrates, decline rapidly as the fruit transpires and the respiratory rate increases [77], thereby converting organic acids to sugars [78]. The 10% GA provided a semipermeable film around the fruit to reduce respiration (gas exchange) that maintained relatively high levels of TA during the breakdown of organic acids, hence delaying the utilization of organic acids [79], whereas the optimum concentration of 10 g L^−1^ of either turmeric or ginger extract was fungistatic against *C. gloeosporioides*, by inhibiting the fungal growth during infection [73] and possibly inhibiting fruit cell wall degrading enzymes [80]. The presence of 10 g L^−1^ crude extract reduced fungal attack by delaying the secretion of ammonia as a buffer from the fungus, thereby reducing dragon fruit tissue alkalinization and increasing TA (decreasing the pH of the fruit) [47].

### 5.3. Efficacy of Formulation on Gas Exchange

No respiration peak and very little ethylene were detected during the early storage time from day 7 to day 16, a phenomenon that describes the nonclimacteric nature of dragon fruit [81]. However, the sudden peak in respiration rate at the end of the storage was due to tissue damage (wounding) caused by a fungal attack [82–84]. This supports an earlier finding by El Ghaouth et al. [82] and Jiang and Li [83], where an increase in respiration rate in strawberries and longan fruit due to disease infection was observed. In this study, a composite coating of 10% GA plus 15 g L^−1^ of ginger and/or 10% GA+10 g L^−1^ of turmeric-coated fruit reduced respiration and to some extent ethylene production. The decrease, which was maintained from day 7 until day 14, could be due to the synergistic effect of 10% GA that modified the internal atmosphere of the fruits, thereby reducing respiration rate and ethylene production while crude extracts at, especially 10 g L^−1^ turmeric or 5 g L^−1^ ginger provided an antifungal barrier against fungal attack.

### 5.4. Efficacy of Formulation on External Attributes and Sensory Evaluation

Contrary to the research by Bordoh et al. [22] who reported discoloration of fruit peel color and unpleasant odor of fruit due to the application of high concentration (10 or 15 g L^−1^) of turmeric and ginger extracts, the incorporation of 10% GA with 10 or 15 g L^−1^ of turmeric or ginger extract markedly gave a glossy external appearance of the fruit. In other words, the original dragon fruit peel color was maintained while the peel odor from ginger and turmeric was reduced when 10% GA was incorporated with crude extract at 10 or 15 g L^−1^. Fruit coated with 10% GA+10 g L^−1^ largely maintained most of the postharvest quality parameters and reduced disease incidence and severity, hence contributing to maintaining the keeping quality, prolonged shelf life, and overall acceptability.

## 6. Conclusion

The present study showed that a composite coating of 10% GA+10 g L^−1^ (for all extracts) or 10% GA+15 g L^−1^ (ginger extract) significantly reduced anthracnose and its severity in dragon fruit. However, this was pronounced when the fruit was coated with 10% GA+10 g L^−1^ of turmeric crude extract. The study also demonstrated that incorporation of 10% GA with ginger extract at low (5 g L^−1^) or high concentrations (10 or 15 g L^−1^) significantly reduced phytotoxicity, contrary to a study by Bordoh et al. [22] who reported phytotoxicity for the above treatments. Additionally, incorporation of plant extract especially at 10 g L^−1^ of turmeric extract with 10% GA delayed fruit ripening and maintained the keeping quality of fruit as this was evident in delayed color changes, high firmness, high TA, reduced respiration rate, and ethylene production. Additionally, fruit coated with 10% GA+10 g L^−1^ of turmeric attained the best overall score in terms of good external appearance, pulp color, aroma, sweetness, texture, and overall acceptability by the panel. We conclude that the incorporation of plant crude extract, especially 10 g L^−1^ of turmeric with 10% GA can serve as a biofungicide in controlling postharvest disease of dragon fruit, reduce possible phytotoxicity, and maintain the postharvest quality of dragon fruit. As an emerging hurdle technology, the incorporation of gum arabic with plant extracts such as turmeric is a safe postharvest treatment (biofungicide) in postharvest management of diseases while maintaining the postharvest quality of fresh produce. This is because gum arabic and turmeric are natural products, are generally regarded as safe, and do not leave any dangerous residual effect on fruits, compared to some commercial synthetic preharvest fungicides like mancozeb. This research will contribute to key advances in food science, food safety and security, and mechanistic aspects of promising emerging food innovative technologies to enhance fruit quality, extend the shelf life, and ultimately reduce postharvest losses of fruits.

## Figures and Tables

**Table 1:** Sensory evaluation of dragon fruits treated with plant extracts at different concentrations incorporated with 10% GA.

Treatment	Sensory quality parameters					
	Appearance	Pulp color	Aroma	Sweetness	Texture	Overall acceptability
Control	2.17a	2.33c	3.67a	1.67d	3.17ab	2.00c
10% GA+ 5 dun	3.33a	3.0b	3.17b	3.16bc	2.67bc	3.17a
10% GA+10 dun	3.33a	3.5ab	3.33b	3.00bc	3.17ab	3.17a
10% GA+5 tur	3.50a	3.67a	3.33a	3.50a	3.33ab	3.50a
10% GA+ 10 tur	3.67a	3.67a	4.17a	4.67a	3.83a	3.83a
10% GA+ 5 gin	3.50a	3.67a	3.33b	3.67bc	2.83bc	3.33a
10% GA+ 10 gin	3.50a	3.50ab	2.83bc	2.50c	3.00abc	3.00a
Sem	0.252	0.2218	0.27	0.287	0.309	0.303
*P* value	0.001	0.001	0.001	0.001	0.021	0.007

Means with different letters in a column are significantly different at *P* < 0.05 using the Fisher unprotected test. Storage conditions; 21 days at 11 ± 2°C, 80% RH (*n* = 42 fruits).

## Data Availability

Data is available on request. Kindly contact the corresponding author.

## Additional Points

*Highlights*. (i) Incorporation of gum arabic (GA) with medicinal plant crude extract at high concentrations reduces phytotoxicity. (ii) GA serves as a carrier for antimicrobial compounds in plant extracts during incorporation. (iii) Composite coating of GA and optimum concentration of turmeric crude extract reduces postharvest anthracnose. (iv) Composite coating of GA and optimum concentration of turmeric crude extract prolongs the shelf life of dragon fruit.
